# A Society in Transition: Presidential Remarks at the 65th Annual SSO Cancer Symposium

**DOI:** 10.1245/s10434-012-2443-y

**Published:** 2012-07-18

**Authors:** James S. Economou

**Affiliations:** Division of Surgical Oncology, University of California, Los Angeles, CA USA

I would like to express to the membership of the Society of Surgical Oncology (SSO) how deeply honored I have been to serve as your 65th president. This was an undeserved and unexpected privilege.

Our specialty is at an inflection point in history occasioned by the unanimous approval of subspecialty board certification by the American Board of Surgery. The 21st century is an exciting time to be a surgical oncologist, with our deeper understanding of cancer biology and a continuous pipeline of molecules that target oncogenes and signaling pathways, and enhance antitumor immunity.

## Role Models in my Academic Career

**Fig. 1:**
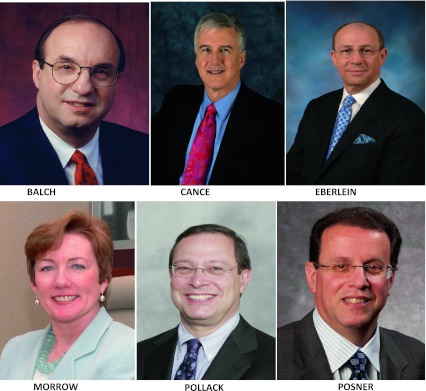
Role models in the SSO: Charles Balch, Bill Cance, Tim Eberlein, Monica Morrow, Raphe Pollock, and Mitch Posner

I want to begin my presidential address by thanking two remarkable surgical leaders: Monica Morrow, who will succeed me as president, and Mitch Posner, my immediate predecessor. Many of you already know what I learned over the last year: Monica is hard-working, highly organized, articulate, and purposeful, as well as a preeminent expert in breast diseases. This society has much to look forward to during her presidency. As I indicated last year when I introduced him to you as president, Mitch is the real deal. He is hard-working, a superior clinical surgeon and investigator, a great leader, and a fundamentally decent person. It has been a privilege to have my term flanked by these two fine surgical leaders (Fig. 1).

I am indebted to many individuals throughout my career, and I feel obligated to quickly acknowledge them. All were role models; some were mentors. A role model is a person regarded as a good example to follow, whose behavior and quality is worth adopting.

Seven faculty at Johns Hopkins had an important impact early in my career: the senior vascular and transplant surgeon Mel Williams, in whose laboratory I first worked; Mack Holmes and Mike Zinner, on whose services I worked as a medical student; and John Cameron, Saul Roseman, and my PhD advisor, Hyun Shin, and his senior colleague, Manfred Mayer. At UCSF, where I completed my surgical training, I had the privilege of being trained by Orlo Clark, Cliff and Karen Deveney, Paul Ebert (who was chair), Nick Feduska, Maurice Galante, David Hohn (who was trained by my father and who later trained me), Frank Lewis, Robert Lim, Carlos Pellegrini, Oscar Salvatierra, Bill Schecter, Don Trunkey, and Larry Way. This was a Pantheon of American Academic Surgery.

In my 26-year career at UCLA, not a few role models stand out: William Longmire, and again Mack Holmes and Mike Zinner, who served as our department chairs; Jean deKernion, Arie Belldegrun and John Glaspy, four basic science colleagues; Judy Gasson, Bill McBride, Mike Phelps, and Owen Witte; and especially my senior colleague in surgical oncology, Fred Eilber, one of the most admired and respected members of the UCLA faculty (Table 1).

**Table 1:** Role models and mentors in James S. Economou’s academic career

Johns Hopkins	
John Cameron	Hyun Shin
Mack Holmes	Mel Williams
Manfred Mayer	Mike Zinner
Saul Roseman	
UCSF	
Orlo Clark	Frank Lewis
Cliff Deveney	Robert Lim
Karen Deveney	Carlos Pellegrini
Paul Ebert	Oscar Salvatierra
Nick Feduska	Bill Schecter
Maurice Galante	Don Trunkey
David Hohn	Larry Way
Frank Lewis	
UCLA	
Arie Belldegrun	William Longmire
Jean deKernion	Bill McBride
Fred Eilber	Mike Phelps
Judy Gasson	Owen Witte
John Glaspy	Mike Zinner
Mack Holmes	

Several leaders in our society played influential roles in my career: Charles Balch, Bill Cance, Tim Eberlein, and Raphe Pollock (also trained by my father) (Fig. 1). For some reason, these four surgical leaders, who have become good friends, always seem to be working in the background to support my career.

**Fig. 2:**
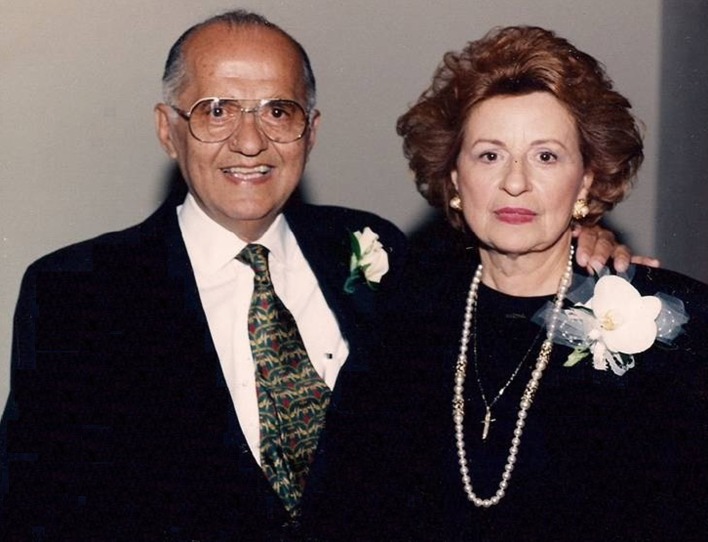
My parents, Steven and Kathryn Economou. This photo was taken on July 11, 1994, on the occasion of a reception, attended by several hundred grateful patients, celebrating the establishment of the Steven G. Economou Chair of Surgery at Rush Medical College

It never hurts if preeminent role models are your parents. Shown here are Steven and Kathryn Economou, the son and daughter of Greek immigrants (Fig. 2). My father was a past member of this society, a surgical educator and scientist, and a surgeon’s surgeon. He and my mother were outstanding role models to three successful children and 12 successful grandchildren.

I finally need to thank my loving and supportive family: my wife, Denice, and my three sons, Steven, Peter, and George, who have been a continuous source of pride in my life.

## Cancer Surgery at the Los Angeles Zoo (Delivered as 65th SSO Presidential Address, March 23, 2012)

I want to describe to you some of the experiences I’ve had as one of the founding members of the Los Angeles Zoo Medical Advisory Board. Throughout my career, I’ve conducted animal research with the goal of developing new therapies that would change the care of human patients. These have all been complex cell- and gene-based therapies. But I’ve had the opportunity over the last 15 years to work with a group of outstanding zoo veterinarians and animal curators using knowledge of human disease and biology as well as the implementation of modern technology to help animals.

**Fig. 3:**
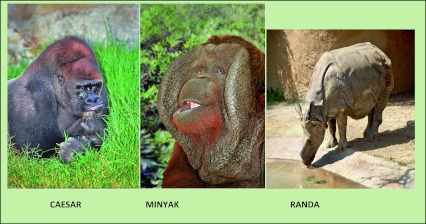
Patients at the Los Angeles Zoo

A video entitled “Cancer Surgery at the Los Angeles Zoo” depicts the presidential address delivered on March 23, 2012 (http://www.surgery.medsch.ucla.edu/economou.wmv). Three surgical operations on large animals at the Los Angeles Zoo are shown: Caesar, a male silverback lowlands gorilla who had a recurrent pleomorphic adenoma of the parotid gland; Minyak, a purebred Borneo orangutan with a recurrent infected laryngeal air sac that required a complete resection as a life-saving operation; and Randa, a single-horned Indian white rhinoceros with recurrent multifocal squamous carcinoma of the horn (Fig. 3).

## A Specialty Society in Transition (Delivered at the 65th SSO Business Meeting, March 24, 2012)

Since its founding just before World War II as the James Ewing Society, the SSO has become the preeminent cancer surgery association of the Western Hemisphere. The SSO has strong and mutually beneficial relationships with sister societies in the Americas, Asia, Australia, Europe, the Middle East, Africa, and the Indian subcontinent.

Founded by a handful of cancer surgeons at Memorial Hospital in New York, the SSO now has a membership of 2,772, which includes surgeons from 52 countries. Over the last several years, attendance at the SSO Annual Cancer Symposium averaged 1,155, of which 22 % were international attendees.

The *Annals of Surgical Oncology,* led from its inception in 1994 by Past President Charles Balch, is now one of the most important and influential surgical journals. Its prestige has enhanced the international outreach of our society. This journal is owned by the Society, is currently published by Springer, and has a 5-year impact factor of 4.412 with a circulation, increasingly international, of approximately 11,000. The volume and quality of submitted manuscripts has been such that serious discussions of founding a sister journal have started.

A seminal event occurred in 2012: the American Board of Medical Specialties unanimously approved the application for a subspecialty certificate in Complex General Surgical Oncology (March 22, 2011) and was ratified by the assembly the next day. The events that culminated in this approval were eloquently described in the 2010 presidential address delivered by Fabrizio Michelassi.^1^ This outcome represented a continuum of SSO leadership beginning over 25 years ago with SSO Past President Charles Balch and overcoming multiple hurdles. Surgical oncology training is now recognized to embody cognitive and practice skills that adequately differentiate it as general surgical subspecialty. Our society needs to recognize many surgical leaders who helped effect this outcome: Russell Berman, B. Mark Evers, Jeffrey Gershenwald, Nathalie M. Johnson, Suzanne Klimberg, Frank R. Lewis Jr., Christopher R. McHenry, Fabrizio Michelassi, Jeff Moley, Carlos Pellegrini, Mitch Posner, Rache Simmons, Douglas Tyler, Selwyn Vickers, and Ronald J. Weigel.

Coinciding with certificate approval has been the explosive growth in the 21st century in our understanding of human cancer biology. This has been occasioned by remarkable advances in cancer genetics and immune biology that have allowed rational development of targeted therapies. These new therapies, which are more effective and have fewer adverse effects, are easier to prescribe. The 21st-century surgical oncologist—with advanced training, both clinical and cognitive—will be a complete oncologist.

The SSO leadership and administration has continued to support the aspirations of its members through a variety of mechanisms. During the Michelassi presidency, Mitch Posner led a strategic planning process at a retreat in Chicago on July 23–24, 2010, that charted the future trajectory of our modern surgical society. These major steps, described by him during his presidential address, have positioned the SSO to become a preeminent global cancer society.^2^ The Posner Strategic Plan recognized the need to create a big tent for all cancer surgeons, to reorganize and reinvigorate its governance, to create an independent administrative organization, and to greatly enhance education and training for surgical oncologists at all stages in their careers.

Under the leadership of President Elect Monica Morrow, a transition committee (Charles Balch, Dan Coit, and Mitch Posner) moved the SSO into an independent administrative structure with new office space, an executive director (Eileen Widmer) who now reports directly to the Executive Council, and direct accountability for finances and performance, all giving the SSO the flexibility to meet rapidly evolving needs.

With the leadership of Richard Alexander, the SSO committee structure was completely overhauled. Twenty-nine committees, all reporting to the Executive Council, are now consolidated into 14, housed in three committee groups (Table 2), with each group overseen by SSO officers. Bylaws were rewritten, committee handbooks completely reworked, and a policy and procedure/leadership manual created. This reorganization has allowed greater oversight, accountability, and improved decision making.

**Table 2:** Current SSO committee structure

Executive/Administrative Group (President/President Elect)
Executive Committee (officers)
Nominating Committee
Constitution and Bylaws Committee
Finance Committee
Education Group (Vice President)
Scientific Program Committee
* Annals of Surgical Oncology* (education function)
Training Committee (Breast Oncology and Surgical Oncology subcommittees)
CME Committee
Educational Products Committee
Disease-site workgroups
Membership Services Group (Secretary, Treasurer)
Membership Committee
Outreach Committee
Fellowship and Research Grant Committee
Technology and Communications Committee
Direct Reports to Executive Council
James Ewing Foundation
*Annals of Surgical Oncology* (editor, publisher)

The Education Committee group, led by Vice President Suzanne Klimberg, was supported by Russ Berman, Jason Fleming, Tari King, Kevin Roggin, Danny Takanishi, and Ron Weigel. They faced the task of meeting—in fact, at times even defining—the educational and training needs of our society membership. Many of the current SSO-approved general surgical oncology training programs will be able to successfully transition to Accreditation Council for Graduate Medical Education (ACGME)-approved programs in complex general surgical oncology. Russ Berman and the Training Committee will assist existing and aspiring programs to meet the high standards of a Board-approved training environment.

After a full-day planning session in Chicago on December 5, 2011, led by Suzanne Klimberg and Ron Weigel, the Education Committee Group recommended, and the SSO Executive Council approved, the creation of a Surgical Oncology Self-assessment Program (SOSAP). SOSAP, modeled after the very successful self-assessment program offered by the American College of Surgeons, to be published electronically as evidence-based, practice-relevant educational products updated every 3 years. It will benefit fellows in training and practicing surgical oncologists at all stages in their careers. Vice President Ron Weigel will be editor in chief and will be supported by members of the SSO Disease Site workgroups.

The Education Committee Group and its member committees introduced several additional innovations this last year. A successful Fellows Institute (Dave Bartlett, Charles Cox, Jill Dietz, Suzanne Klimberg, and Charles Scoggins) was offered to 31 surgical oncology fellows and 48 breast oncology fellows in August 2011, providing hands-on laparoscopic surgical training. This will be reprised in November 2012 with more customized tracks in breast, general surgical oncology, and hepatobiliary techniques. Danny Takanishi’s Continuing Medical Education Committee members maintained and enhanced SSO accreditation status, improved annual meeting evaluations instruments, and helped pilot, with the Maintenance of Certification Committee, an MOC Part 2 activity. The Program Committee (Jason Fleming, Tari King) organized a well-received 65th Annual Cancer Symposium to which a record number of abstracts were submitted (*n* = 648) and in which various educational enhancements were tested.

Finally, this year, the James Ewing Foundation, under the leadership of Past President Bill Cance, was established as the sole fund-raising arm of the SSO. It is completely aligned with the mission of the SSO, ensures corporate compliance, and houses the Corporate Relations Committee.

In summary, much work over the last year has positioned our society to prosper in the 21st century of molecular oncology and to provide international leadership in surgical oncology education and training, all with the goal of better serving our patients.
